# The role of postoperative blood pressure management in early postoperative hemorrhage in awake craniotomy glioma patients

**DOI:** 10.1007/s10143-024-02661-0

**Published:** 2024-08-22

**Authors:** Matthias Demetz, Aleksandrs Krigers, Rodrigo Uribe-Pacheco, Daniel Pinggera, Julia Klingenschmid, Claudius Thomé, Christian F. Freyschlag, Johannes Kerschbaumer

**Affiliations:** Department of Neurosurgery, Medical University of Innsbruck, Anichstr. 35, Innsbruck, AT-6020 Austria

**Keywords:** Awake craniotomy, Postoperative hemorrhage, Blood pressure management, Neuro-oncology

## Abstract

Postoperative hemorrhage can severely affect the patients’ neurological outcome after awake craniotomy. Higher postoperative blood pressure can increase the risk of postoperative hemorrhage. The aim of this study was to investigate the role of postoperative blood pressure and other common radiological and epidemiological features with the incidence of postoperative hemorrhage. In this retrospective analysis, we included patients who underwent awake surgery at our institution. We assessed the blood pressure both intra- and postoperatively as well as the heart rate for the first 12 h. We compared a cohort with postoperative hemorrhage, who required further treatment (surgical revision or intravenous antihypertensive therapy), with a cohort with no postoperative hemorrhage. We included 48 patients with a median age of 39 years. 9 patients (19%) required further treatment due to postoperative hemorrhage, which was surgery in 2 cases and intensive blood pressure measurements in 7 cases. However, with early treatment, no significant difference in Performance scores at follow-up could be found. Patients with postoperative hemorrhage showed significantly higher postoperative systolic blood pressure during the hours 3–12 (*p* < 0.05) as well as intraoperatively throughout the procedure (*p* < 0.05). In ROC and Youden Test, a strong impact of systolic blood pressure over 140mmHg during the early postoperative course could be shown. Postoperative hemorrhage is a rare but possible complication in awake surgery glioma patients. To avoid postoperative hemorrhage, treating physicians should aim strictly on systolic blood pressure of under 140mmHg for the postoperative course.

## Introduction

The surgical management of gliomas has proven to be the most effective initial treatment to improve outcomes [1–4]. In eloquent gliomas, awake surgery with real-time mapping of eloquent brain areas could be established allowing maximal safe tumor [5, 6]. ostoperative complications following awake craniotomy, such as postoperative hemorrhage, represent a significant concern in the management of glioma patients [7]. Elevated blood pressure during and after surgery can increase the risk of postoperative hemorrhage, potentially leading to detrimental consequences for the patient, including prolonged hospital stays, compromised neurological function, and even mortality [8–11]. Conversely, inadequate blood pressure management may jeopardize cerebral perfusion, leading to ischemia, infarction, and might also result in neurological deficits [12, 13]. The challenge, therefore, lies in optimizing blood pressure control during and after awake craniotomy to minimize both the risk of bleeding and the potential for postoperative neurological deficits. This requires continuous blood pressure measurement with some patients necessitating anti-hypertensive treatments transiently after craniotomy for glioma resection, which can usually only be provided by invasive blood pressure measurements in intermediate or intensive care units [14–16].

In recent years, there has been a growing interest in understanding the relationship between blood pressure management and postoperative complications in glioma patients. As previously described, comorbidities as well as fluctuations of blood pressure can significantly influence the postoperative morbidity in glioblastoma (GBM) patients [17, 18]. However, standardized blood pressure goals after a craniotomy for glioma resection are not well-defined and demonstrate the need for further studies [16, 19, 20]. Data on the impact of blood pressure management, other radiological and neuro-oncological parameters like residual tumor volume as potential source of bleeding, preoperative hypertension and patient age on the occurrence of postoperative complications like hemorrhage are limited. Furthermore, patients undergoing awake craniotomy are subjected to higher stress levels compared to those under general anesthesia, which can potentially increase the risk of postoperative hemorrhage. The perioperative stress in awake craniotomy patients caused by potentially higher pain levels, the desire to perform well, and the unfamiliar position and setting, might lead to higher blood pressure and, therefore, an increased risk of postoperative hemorrhage.

This study therefore aimed to investigate the role of various preoperative epidemiological, radiological and neuro-oncological risk factors as well as the role of postoperative blood pressure management in the development of early postoperative hemorrhage. We furthermore aimed to identify blood pressure goals after craniotomy to minimize the risk of postoperative hemorrhage.

## Material & methods

In this study, adult patients (≥ 18 years at the time of surgery) who underwent first awake craniotomy for intracranial glioma at the authors’ institution between January 2016 and March 2023 were retrospectively reviewed. Patients were considered for awake craniotomy if they had a supratentorial lesion located within or close to regions presumed to harbor language and/or sensorimotor function on preoperative MRI. Epidemiological and clinical data could be retrieved from the prospective institutional neuro-oncological database. Histological diagnosis was assessed considering the revised 4th WHO grading system of central nervous system tumors of 2016 and 2021 respectively [21, 22], depending on the date of diagnosis. Pre-treated (partial resection, recurrence and previously biopsied) patients as well as bi- and trilanguage tested and non-native speakers (both due to the longer surgical time with multiple rounds of testing) were excluded from this study.

Awake craniotomy was performed as standard of care at the authors’ institution in all eligible patients with eloquent tumors affecting language functions according to international guidelines. Patients were seen postoperatively at three- or six-months intervals, depending on the WHO grade and molecular characteristics of the glioma. Patients’ general condition was assessed using the Karnofsky Performance Score (KPS) pre- and postoperatively.

IDH1-mutation in the R132H position was assessed using immunohistochemistry (IHC) and in case of a negative result DNA sequencing for patients under 40 years was performed to confirm wildtype IDH1 and IDH2 status. Expression of nuclear alpha thalassemia mental retardation X-linked (ATRX), epidermal growth factor receptor (EGFR) and MIB-1 as proliferation marker were tested by IHC. If IDH, ATRX and EGFR were not tested routinely and tissue was available for further analysis, the parameters were re-assessed for this trial.

Awake craniotomy was performed using a sleep-awake-awake or completely awake protocol with sedoanalgesia. Patients underwent routine neuropsychological testing before surgery assessing various cognitive parameters like semantic and figural memory as well as executive functions and language testing.

All our patients were postoperatively transferred to the Neurosurgical Intensive Care Unit (ICU) for further observation for 18–24 hours. All patients were monitored for heart rate, neurological status and blood pressure using blood pressure cuff or arterial line and hypertension was consequently treated with different antihypertensive drugs and pain medication. Postoperative MRI was performed within 48 hours. In case of severe hemorrhage surgical evacuation or prolonged ICU observation were considered, depending on the mass effect and clinical presentation of the patient. Postoperative hemorrhage’ was defined as the presence of visual blood in the resection cavity or the surrounding brain tissue observed on postoperative imaging. Furthermore, we classified treatment-requiring hemorrhage as extensive bleeding causing mass effect accompanied by new neurological deficits in the patient.

Magnetic resonance imaging (MRI) including T1-weighted Gadolinum-contrasted as well as native T1, T2, FLAIR and DWI sequences were performed preoperatively and early postoperatively as the standard of care [23]. Tumor volume was manually assessed using segmentation in ITK-SNAP software (v.3.8.0 for Mac OS, UPenn and UNC dev.) in T1 CE as well as native T1, T2, FLAIR (fluid attenuated inversion recovery) and DWI (diffusion weighted imaging) sequences [24].

Data evaluation was performed using IBM SPSS Statistics (IBM SPSS Statistics for Mac OS, Version 27.0. Armonk, NY: IBM Corp.). Scale variables were determined with T-tests and shown as mean with standard deviation (SD) in case of normal distribution or with Mann-Whitney U-test and shown as median with interquartile range (IqR), if normal distribution was not achieved. Binominal pairs were compared using Chi-squared test. The mean estimated PFS and OS times were assessed with Kaplan–Meier processing and analyzed with LogRank test. We used Cox regression assessment to reveal hazard ratios (HR) for oncological progression or death. The α value was defined as 0.05, and 95% confidence intervals were constructed.

This study was approved by the institutional ethics committee of Medical University of Innsbruck (1333/2021). This study was conducted in accordance with the ethical standards as laid down in the 1964 Declaration of Helsinki and its later amendments.

## Results

48 patients (28 male, 20 female) with a median age of 39 years (Interquartile Range (IqR) 29–49, absolute range 20–74) at the time of surgery could be included in this study. Median preoperative KPS amounted to 100 (IqR 90–100), indicating a low burden of disease in our patients. The high KPS could be maintained at first follow up with a median KPS of 100 (IqR 90–100) after three months.

Most frequent tumor location was the temporal lobe with 26 patients, the frontal lobe was affected in 20 patients. Mean preoperative tumor volumes in various MRI sequences are shown in Table 1.

**Table 1:** Mean preoperative and postoperative tumor volumes in awake craniotomy patients as well as median extent of resection after surgery

MRI sequence	Mean preoperative volume (SD±)	Mean postoperative volume (SD±)	Median extent of resection (IqR)
T1 CE	6.5 cm^3^ (10.5)	0.2 cm^3^ (0.6)	100% (96.8–100)
T2	42.3 cm^3^ (35.4)	10.5 cm^3^ (3.1)	90.6% (73.5–100)
FLAIR	57.9 cm^3^ (47.7)	15.5 cm^3^ (3.4)	93.6% (70.4–100)

Nine patients (19%) demonstrated a previous history of arterial hypertension, of whom 8 were on antihypertensive treatment. Only one patient (2%) had a preoperative history of coronary artery disease. The most frequent preoperative symptom was epilepsy in 35 cases (73%). 7 patients (15%) harbored incidental findings.

The most frequent histological diagnosis was a diffuse glioma CNS WHO°2, with 14 patients (29%). The diagnosis of an anaplastic glioma CNS WHO°3 was confirmed in 16 cases (33%) and 38% of the cases were diagnosed as GBM (CNS WHO°4). IDH-1 showed a mutation in 64% and IDH-1 wildtype in 36%, EGFR was expressed in 74% and showed no expression in 26% while ATRX was expressed in 59% and lost in 41% of our patients.

In the postoperative MRI, we observed a small, non-significant rim of blood in 14 patients (29%) who remained asymptomatic and did not necessitate additional intervention. In nine cases of our patient cohort (19%), further treatment was deemed necessary due to postoperative hemorrhage to prevent potential neurological deterioration. Only 2 (4% of the cohort) of those 9 patients required revision surgery, the other patients were treated conservatively with prolonged observation and stay at our ICU for intravenous antihypertensive therapy.

Mean follow-up (FU) was 48 months (SD ± 43) in our cohort. Kaplan-Meier analysis revealed an estimated mean overall survival (OS) of 90 months (SD ± 10). Due to the low malignant course of especially lower grade gliomas (LGG), OS was not reached in all patients.

In the Cox Regression analysis, OS was significantly influenced by age (HR per year 1.063, *p* = 0.003), WHO grade (HR per grade 2.925, *p* < 0.001), preoperative KPS (HR per 10-point increase 0.916, *p* = 0.013), KPS at first FU (HR per 10-point increase 0.958, *p* = 0.012), in case of preoperative history of arterial hypertension (HR 4.062, *p* = 0.006) and postoperative hemorrhage (HR 69.86, *p* = 0.001). Regarding the volumetric analysis, preoperative tumor volume in T2 sequence (*p* = 0.006) and the extent of resection (*p* = 0.030) showed a significant impact on OS in Cox Regression. No significant influence on OS could be shown for gender (*p* > 0.05), molecular features like ATRX and EGFR (*p* > 0.05) and tumor location (*p* > 0.05).

Logistic regression revealed no significant relationship between preoperative tumor volume and postoperative bleeding necessitating surgical revision (*p* > 0.05).

**Fig. 1:**
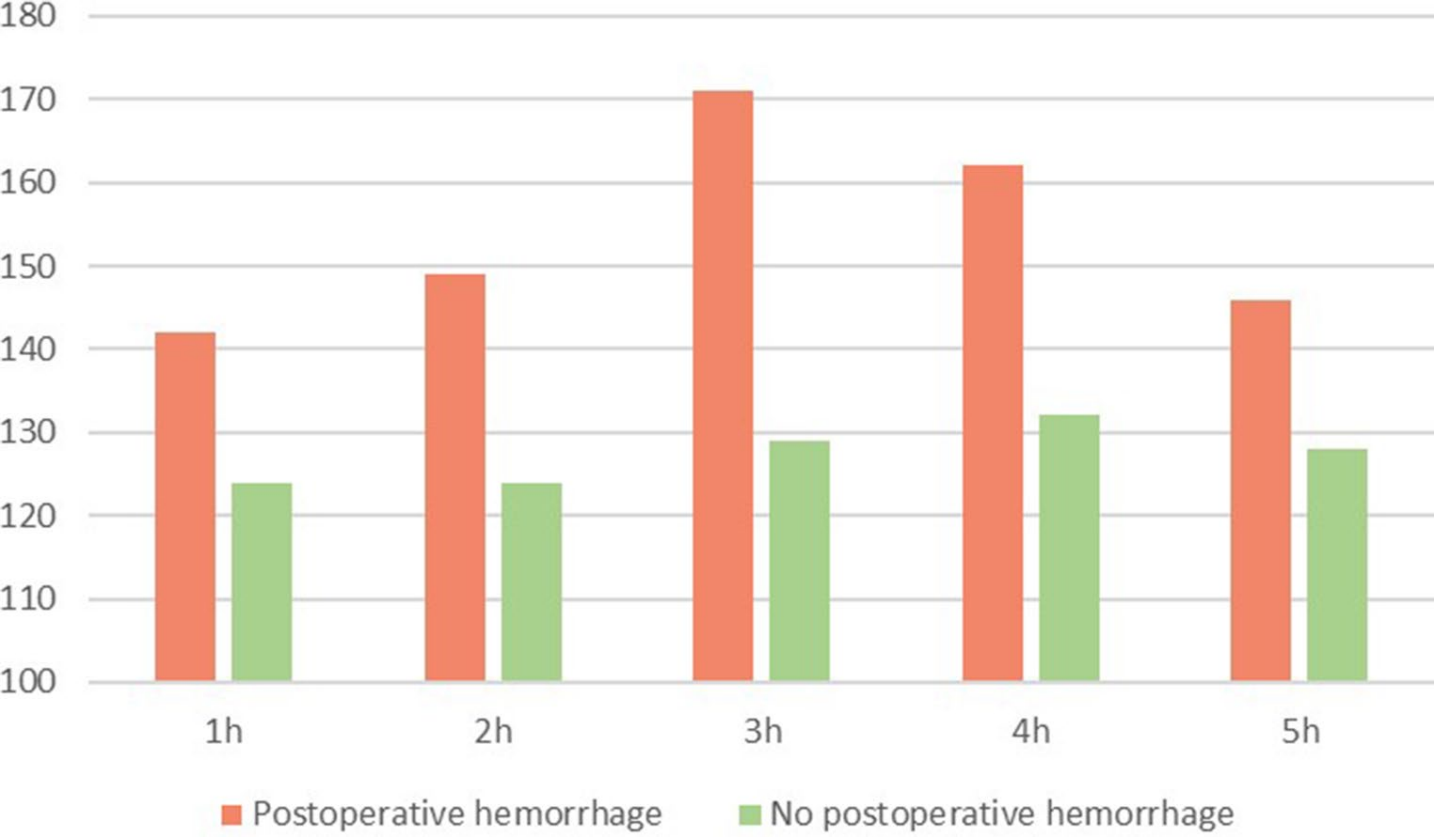
Mean intraoperative systolic blood pressure in mmHg during awake craniotomy and glioma resection

Differences in mean intraoperative systolic blood pressure between patients with postoperative hemorrhage and no postoperative hemorrhage are shown in Fig. 1.

Patients with postoperative hemorrhage showed a mean time of 172 min (SD ± 95) of systolic blood pressure above 140 mmHg during surgery, while patients with no postoperative hemorrhage showed a shorter mean time of 38 min (SD ± 47).

**Fig. 2:**
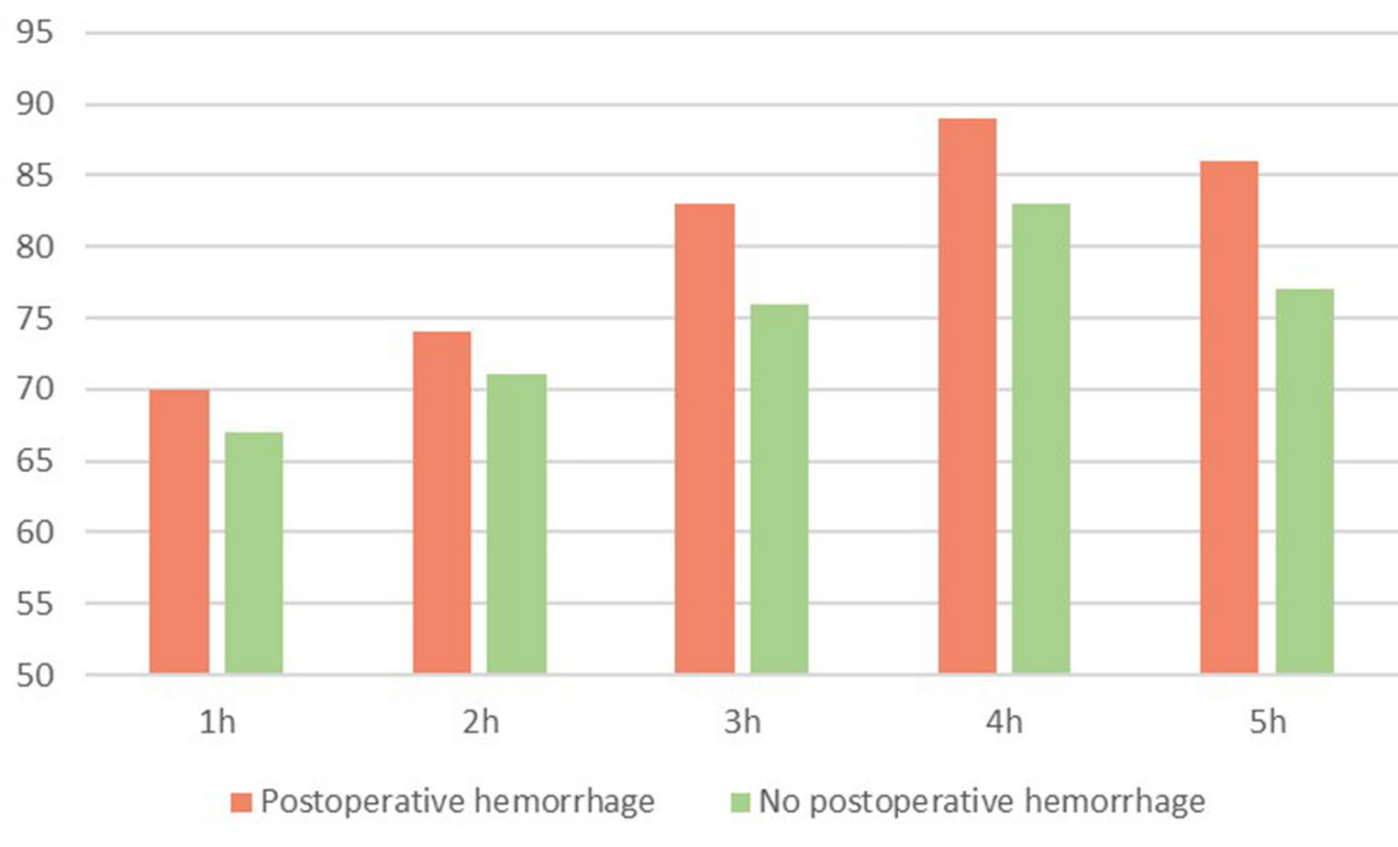
Mean intraoperative heart rate in bpm during awake craniotomy and glioma resection

Differences in mean intraoperative heart rate in beats per minute (bpm) between patients with postoperative hemorrhage and no postoperative hemorrhage are shown in Fig. 2.

Patients with postoperative hemorrhage showed a mean time of 55 min (SD ± 71) of heart rate over 100 bpm during surgery, while patients with no postoperative hemorrhage showed a mean time of 31 min (SD ± 37).

**Fig. 3:**
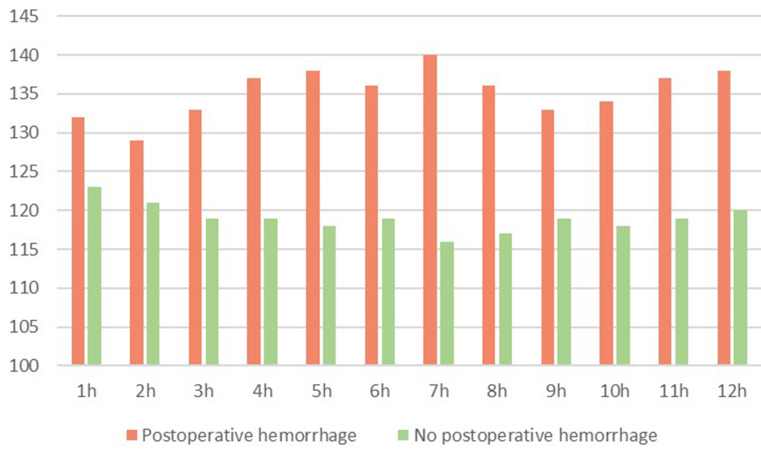
Mean postoperative systolic blood pressure in mmHg after awake craniotomy and glioma resection

Differences in mean postoperative systolic blood pressure between patients with postoperative hemorrhage and no postoperative hemorrhage are shown in Fig. 3.

No significant differences in mean postoperative heart rate between patients with postoperative hemorrhage and no postoperative hemorrhage could be found (*p* > 0.05).

**Fig. 4:**
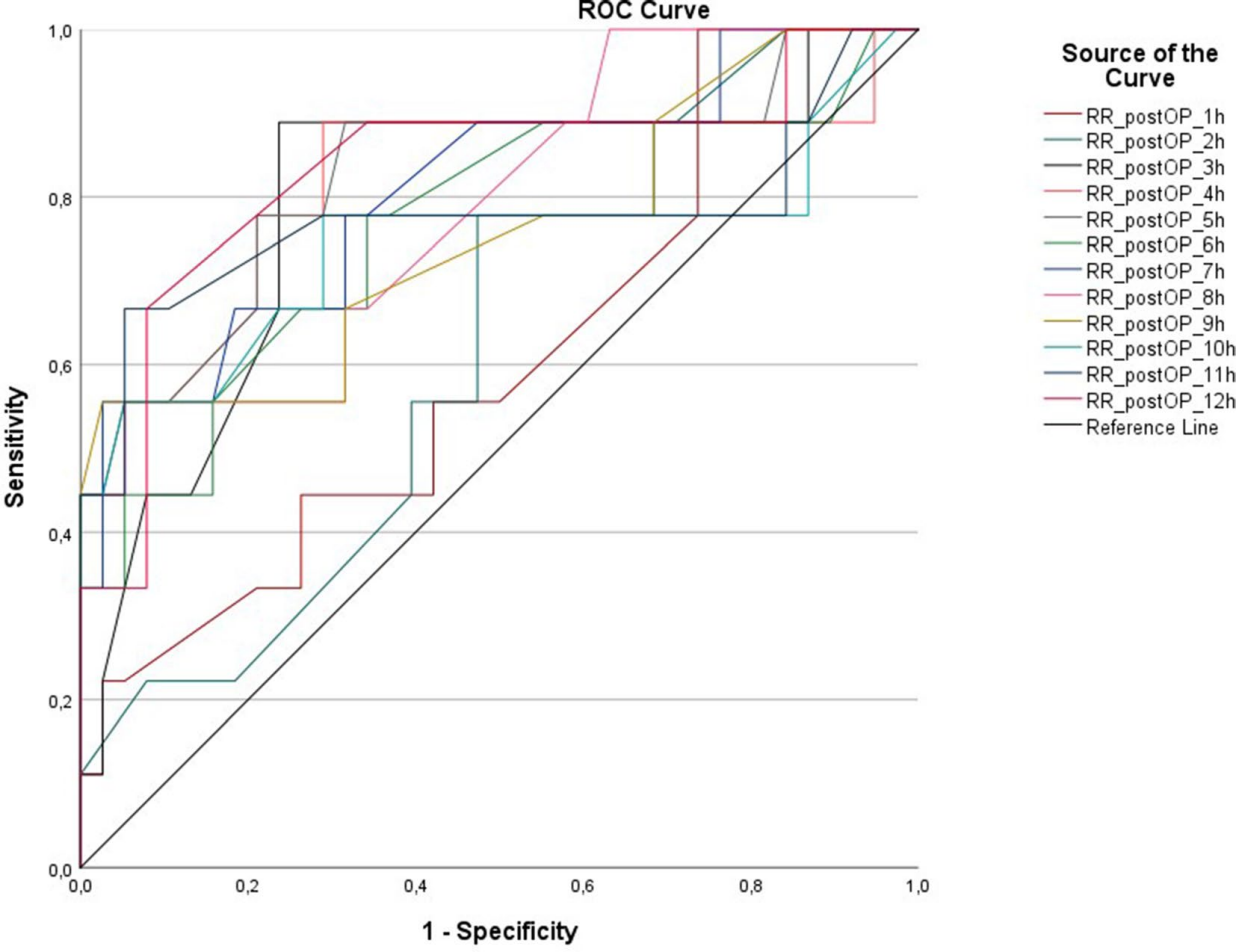
ROC analysis showed a significant difference for postoperative hemorrhage for the postoperative systolic blood pressure during the hours 3–12

**Fig. 5:**
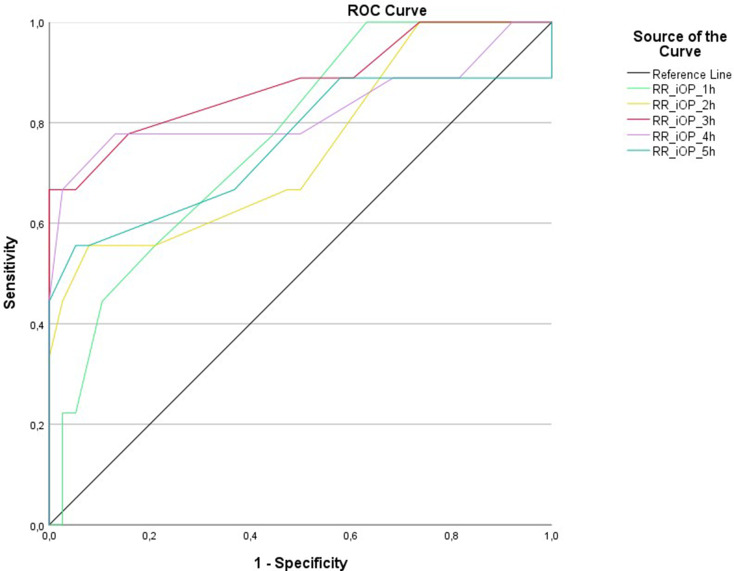
ROC analysis showed a significant difference for postoperative hemorrhage for the intraoperative systolic blood pressure during the whole procedure (hours 1–5)

ROC analysis for postoperative hemorrhage necessitating further intervention revealed significant differences between systolic blood pressure of the two cohorts for the postoperative hours 3 (*p* = 0.006), 4 (*p* = 0.004), 5 (*p* = 0.002), 6 (*p* = 0.016), 7 (*p* = 0.004), 8 (*p* = 0.008), 9 (*p* = 0.019), 10 (*p* = 0.025), 11 (*p* = 0.011) and 12 (*p* = 0.002). Regarding the intraoperative systolic blood pressure, ROC analysis showed a significant difference for all of the hours 1–5 (*p* = 0.014, *p* = 0.022, *p* < 0.001, *p* = 0.003, *p* = 0.018 respectively). No significant difference could be found for the systolic pressure during the first two postoperative hours and for the intraoperative as well as the postoperative heart rate. Results of the ROC analysis are shown in Figs. 4 and 5.

However, postoperative hemorrhage was not translated into a declined KPS at three months follow-up in our analyses, as no significant difference between the two cohorts was found (*p* > 0.05).

The results of the Youden’s index are shown in Table 2.

**Table 2:** Youden’s index showed systolic blood pressure results of approximately 120–125 for the first 6 postoperative hours, but increased for the postoperative hours 7–12 with results of approximately 135–140

Postoperative hour	Systolic blood pressure (mmHg)
1	116–118
2	119–121
3	124–126
4	120–122
5	119–121
6	121–123
7	139–141
8	134–136
9	138–140
10	137–139
11	132–134
12	129–131

## Discussion

In this retrospective study, we reported treatment-requiring early postoperative hemorrhage in 19% of the patients after awake surgery for glioma, supporting the existing literature on the risk of adverse events after awake craniotomy [25–27]. Patients with postoperative treatment-requiring hemorrhage showed both significantly higher intraoperative and postoperative systolic blood pressure compared to patients with no postoperative hemorrhage. ROC analysis and Youden Test revealed a significantly lower risk for postoperative hemorrhage in case of a rigid systolic blood pressure regime of a maximum of 140 mmHg, with even lower results during the first 6 h. Our data therefore suggest a consequent blood pressure management with a maximum of systolic 140 mmHg both intra- and postoperatively to minimize the risk of postoperative hemorrhage. However, further prospective multicenter trials are needed to define standardized blood pressure goals to minimize postoperative morbidity.

Early postoperative hemorrhage is a feared complication in the postoperative course after glioma surgery that might cause neurological deficits and morbidity in often young patients [8–10]. Patients undergoing awake craniotomy are at increased risk of blood pressure fluctuations due to the increased stress level during surgery [28, 29]. However, standardized blood pressure goals after awake craniotomy for intracranial neoplasms are not well-defined.

Our study revealed a higher incidence of postoperative hemorrhage necessitating treatment compared to previous publications [25, 30]. However, it is noteworthy that our study accounted for non-surgical measures such as intravenous antihypertensive therapy, a factor not typically assessed in prior research. By considering a broader spectrum of interventions beyond surgical revisions, we gained a more comprehensive understanding of postoperative hemorrhage management. Moreover, our findings align with existing literature when considering the low number of surgical revisions required in our cohort due to mass effect. This consistency underscores the reliability of our results and suggests that our approach to evaluating postoperative hemorrhage, inclusive of both surgical and conservative measures, contributes valuable insights to the existing body of knowledge in this field.

Our proactive approach to early intervention in cases of postoperative hemorrhage aimed to preserve the high preoperative performance status of our patients and prevent neurological deterioration. Our prompt initiation of treatment proved highly effective, as evidenced by our findings, which showed no significant difference in KPS after three months between patients who experienced postoperative hemorrhage and those who did not. This suggests that despite encountering postoperative hemorrhage, timely intervention effectively mitigated potential negative impacts on patients’ functional outcomes. However, overall survival was significantly influenced by postoperative hemorrhage in our series. The impact of postoperative bleeding on survival could be mediated by its potential to cause new functional deficits and complications that might contribute to earlier death, but this may not be reflected in the KPS scores at the initial follow-up. The significant influence of postoperative hemorrhage on overall survival underscores the critical importance of minimizing this complication to improve patient outcomes.

Our findings reveal a significant correlation between elevated systolic blood pressure during the critical postoperative hours and an increased risk of hemorrhage. Interestingly, our data demonstrated that especially 3 to 12 h after termination of surgery our patients developed an increase in blood pressure. Consequently, the risk of post-operative bleeding also increases significantly in these hours. This could be due to the decreasing sedoanalgesia, which leads to increased pain and increased vigilance. This temporal association suggests a window of vulnerability, in which hemodynamic factors may play a crucial role in the development of hemorrhagic complications [31]. Treatment with pain medication as well as antihypertensive medication should be considered to reach the proposed blood pressure goals and minimize the risk of hemorrhage.

A lower systolic blood pressure in particular during the hours 3–6 should be considered. However, the target range of 120–125 mmHg should be applied judiciously, taking into account the potential risks associated with aggressive antihypertensive treatment like renal failure and ischemia as known possible risk factors of hypotension especially in elderly patients [32, 33]. Considering our findings, the lower systolic target of 120 mmHg represents an ideal goal, the secondary systolic target of 140 mmHg provides a more attainable alternative, especially in situations where achieving the lower target may pose undue risks or practical difficulties.

As previously shown, postoperative hemorrhage might cause significant morbidity and mortality in the often-young patients undergoing awake surgery for gliomas [34, 35]. Our data substantiate the relevance of this complication by revealing a significant impact on overall survival. The imperative to avoid postoperative hemorrhage is grounded not only in the preservation of immediate postoperative outcomes but also in safeguarding the long-term survival, as previously shown in the literature [17, 36]. Our study contributes valuable insights supporting the establishment of a rigid systolic blood pressure limit of 140 mmHg during the early postoperative course. Building upon previous research by Young et al., [16, 19, 20] our data reinforces the notion that maintaining blood pressure within this threshold might be conducive to favorable outcomes in patients undergoing awake glioma surgery.

Contrary to expectations, our data reveal that residual tumor volume did not exert a significant impact on the occurrence of postoperative hemorrhage. While residual tumor volume has historically been considered a potential risk factor due to high vascularization of especially high malignant neoplasms [37, 38], our study challenges this notion, suggesting that other variables like hypertension may play a more pivotal role in determining the likelihood of postoperative hemorrhage.

Contrary to anticipated connections between extracranial cardiovascular dynamics and hemorrhagic complications [39], our study finds that both intra- and postoperative heart rates exhibit no significant impact on the occurrence of postoperative hemorrhage. While heart rate has been traditionally considered a potential indicator of vascular stress [40], heart rate does not seem to play a crucial role in the occurrence of postoperative hemorrhage after awake glioma surgery. However, unlike heart rate, systolic blood pressure emerges as a crucial factor, demonstrating a significant association with the occurrence of hemorrhagic complications.

While our study provides valuable insights into the complex dynamics of awake glioma surgery, several limitations have to be mentioned. Future studies with various multicentric cohorts are warranted to validate and extend the applicability of our observations. We focused predominantly on early hemorrhagic events and blood pressure values within a specific timeframe, restricting our ability to draw comprehensive conclusions about the long-term management of blood pressure, especially beyond the immediate postoperative period. Additionally, our study cohort comprised predominantly young and fit individuals, reflecting a specific demographic profile. The applicability of our findings to frailer cohorts, therefore, remains uncertain, necessitating tailored investigations specific to elderly neuro-oncological patients undergoing similar surgical interventions.

## Conclusion

Postoperative hemorrhage is a possible complication in awake surgery glioma patients. To avoid postoperative hemorrhage, treating physicians should aim strictly on systolic blood pressure of under 140 mmHg for the postoperative course, considering even a lower target during the first six hours. With early intervention, no significant differences could be found during follow-up.

## Data Availability

No datasets were generated or analysed during the current study.

## References

[1] Felsberg J, Rapp M, Loeser S et al (2009) Prognostic significance of molecular markers and extent of resection in primary glioblastoma patients. Clin Cancer Res 15:6683–6693. 10.1158/1078-0432.CCR-08-2801 19861461 10.1158/1078-0432.CCR-08-2801

[2] Sanai N, Polley M-Y, McDermott MW et al (2011) An extent of resection threshold for newly diagnosed glioblastomas. J Neurosurg 115:3–8. 10.3171/2011.2.JNS10998 21417701 10.3171/2011.2.jns10998

[3] Bloch O, Han SJ, Cha S et al (2012) Impact of extent of resection for recurrent glioblastoma on overall survival. J Neurosurg 117:1032–1038. 10.3171/2012.9.JNS12504 23039151 10.3171/2012.9.JNS12504

[4] Brown TJ, Brennan MC, Li M et al (2016) Association of the extent of Resection with Survival in Glioblastoma. JAMA Oncol 2:1460. 10.1001/jamaoncol.2016.1373 27310651 10.1001/jamaoncol.2016.1373 PMC6438173

[5] Hervey-Jumper SL, Li J, Lau D et al (2015) Awake craniotomy to maximize glioma resection: methods and technical nuances over a 27-year period. J Neurosurg 123:325–339. 10.3171/2014.10.JNS141520 25909573 10.3171/2014.10.JNS141520

[6] Gogos AJ, Young JS, Morshed RA et al (2020) Awake glioma surgery: technical evolution and nuances. J Neurooncol 147:515–524. 10.1007/s11060-020-03482-z 32270374 10.1007/s11060-020-03482-z

[7] Di Carlo DT, Cagnazzo F, Anania Y et al (2020) Post-operative morbidity ensuing surgery for insular gliomas: a systematic review and meta-analysis. Neurosurg Rev 43:987–997. 10.1007/s10143-019-01113-4 31098791 10.1007/s10143-019-01113-4

[8] Seifman MA, Lewis PM, Rosenfeld JV, Hwang PYK (2011) Postoperative intracranial haemorrhage: a review. Neurosurg Rev 34:393–407. 10.1007/s10143-010-0304-3 21246389 10.1007/s10143-010-0304-3

[9] Basali A, Mascha EJ, Kalfas I, Schubert A (2000) Relation between Perioperative Hypertension and Intracranial Hemorrhage after Craniotomy. Anesthesiology 93:48–54. 10.1097/00000542-200007000-00012 10861145 10.1097/00000542-200007000-00012

[10] Ahmadipour Y, Kaur M, Pierscianek D et al (2019) Association of Surgical Resection, disability, and survival in patients with Glioblastoma. J Neurol Surg Cent Eur Neurosurg 80:262–268. 10.1055/s-0039-1685170 10.1055/s-0039-1685170 30965373

[11] Saunders CN, Cornish AJ, Kinnersley B et al (2019) Lack of association between modifiable exposures and glioma risk: a mendelian randomisation analysis. Neuro Oncol. 10.1093/neuonc/noz209 10.1093/neuonc/noz209 PMC7442418 31665421

[12] Gempt J, Krieg SM, Hüttinger S et al (2013) Postoperative ischemic changes after glioma resection identified by diffusion-weighted magnetic resonance imaging and their association with intraoperative motor evoked potentials. J Neurosurg 119:829–836. 10.3171/2013.5.JNS121981 23829818 10.3171/2013.5.JNS121981

[13] Gempt J, Förschler A, Buchmann N et al (2013) Postoperative ischemic changes following resection of newly diagnosed and recurrent gliomas and their clinical relevance. J Neurosurg 118:801–808. 10.3171/2012.12.JNS12125 23373806 10.3171/2012.12.JNS12125

[14] Perez CA, Stutzman S, Jansen T et al (2020) Elevated blood pressure after craniotomy: a prospective observational study. J Crit Care 60:235–240. 10.1016/j.jcrc.2020.08.013 32942161 10.1016/j.jcrc.2020.08.013

[15] Lombardi G, Barresi V, Castellano A et al (2020) Clinical management of diffuse low-Grade Gliomas. Cancers (Basel) 12:3008. 10.3390/cancers12103008 33081358 10.3390/cancers12103008 PMC7603014

[16] Young JS, Morshed RA, Hervey-Jumper SL, Berger MS (2023) The surgical management of diffuse gliomas: current state of neurosurgical management and future directions. Neuro Oncol 25:2117–2133. 10.1093/neuonc/noad133 37499054 10.1093/neuonc/noad133 PMC10708937

[17] Liu W, Qdaisat A, Yeung J et al (2019) The Association between Common Clinical characteristics and postoperative morbidity and overall survival in patients with Glioblastoma. Oncologist 24:529–536. 10.1634/theoncologist.2018-0056 30049883 10.1634/theoncologist.2018-0056 PMC6459250

[18] Zhang Y, Ji P, Wang S et al (2022) Early unplanned Reoperation after Glioma Craniotomy: incidence, predictor and process improvement. Front Oncol 12. 10.3389/fonc.2022.898873 10.3389/fonc.2022.898873 PMC9121807 35600362

[19] Anderson CS, Heeley E, Huang Y et al (2013) Rapid blood-pressure lowering in patients with Acute Intracerebral Hemorrhage. N Engl J Med 368:2355–2365. 10.1056/NEJMoa1214609 23713578 10.1056/NEJMoa1214609

[20] Hanak BW, Walcott BP, Nahed BV et al (2014) Postoperative intensive care unit requirements after Elective Craniotomy. World Neurosurg 81:165–172. 10.1016/j.wneu.2012.11.068 23182731 10.1016/j.wneu.2012.11.068 PMC3596491

[21] Louis DN, Perry A, Wesseling P et al (2021) The 2021 WHO classification of tumors of the Central Nervous System: a summary. Neuro Oncol 23:1231–1251. 10.1093/neuonc/noab106 34185076 10.1093/neuonc/noab106 PMC8328013

[22] Louis DN, Perry A, Reifenberger G et al (2016) The 2016 World Health Organization Classification of Tumors of the Central Nervous System: a summary. Acta Neuropathol 131:803–820. 10.1007/s00401-016-1545-1 27157931 10.1007/s00401-016-1545-1

[23] Freyschlag CF, Krieg SM, Kerschbaumer J et al (2018) Imaging practice in low-grade gliomas among European specialized centers and proposal for a minimum core of imaging. J Neurooncol 139:699–711. 10.1007/s11060-018-2916-3 29992433 10.1007/s11060-018-2916-3 PMC6132968

[24] Yushkevich PA, Piven J, Hazlett HC et al (2006) User-guided 3D active contour segmentation of anatomical structures: significantly improved efficiency and reliability. NeuroImage 31:1116–1128. 10.1016/j.neuroimage.2006.01.015 16545965 10.1016/j.neuroimage.2006.01.015

[25] Takami H, Venkatraghavan L, Bernstein M (2022) Perioperative factors affecting Readmission after Awake Craniotomy: analysis of 609 consecutive cases. World Neurosurg 158:e476–e487. 10.1016/j.wneu.2021.11.010 34800731 10.1016/j.wneu.2021.11.010

[26] Lonjaret L, Guyonnet M, Berard E et al (2017) Postoperative complications after craniotomy for brain tumor surgery. Anaesth Crit Care Pain Med 36:213–218. 10.1016/j.accpm.2016.06.012 27717899 10.1016/j.accpm.2016.06.012

[27] Kwinta BM, Myszka AM, Bigaj MM et al (2021) Intra- and postoperative adverse events in awake craniotomy for intrinsic supratentorial brain tumors. Neurol Sci 42:1437–1441. 10.1007/s10072-020-04683-0 32808173 10.1007/s10072-020-04683-0 PMC7955997

[28] Costello TG, Cormack JR (2004) Anaesthesia for awake craniotomy: a modern approach. J Clin Neurosci 11:16–19. 10.1016/j.jocn.2003.09.003 14642359 10.1016/j.jocn.2003.09.003

[29] Dilmen OK, Akcil EF, Oguz A et al (2017) Comparison of conscious sedation and asleep-awake-asleep techniques for Awake Craniotomy. J Clin Neurosci 35:30–34. 10.1016/j.jocn.2016.10.007 27771234 10.1016/j.jocn.2016.10.007

[30] Groshev A, Padalia D, Patel S et al (2017) Clinical outcomes from maximum-safe resection of primary and metastatic brain tumors using awake craniotomy. Clin Neurol Neurosurg 157:25–30. 10.1016/j.clineuro.2017.03.017 28384595 10.1016/j.clineuro.2017.03.017

[31] Abaziou T, Tincres F, Mrozek S et al (2020) Incidence and predicting factors of perioperative complications during monitored anesthesia care for awake craniotomy. J Clin Anesth 64:109811. 10.1016/j.jclinane.2020.109811 32320919 10.1016/j.jclinane.2020.109811

[32] Bijker JB, Persoon S, Peelen LM et al (2012) Intraoperative Hypotension and perioperative ischemic stroke after general surgery. Anesthesiology 116:658–664. 10.1097/ALN.0b013e3182472320 22277949 10.1097/ALN.0b013e3182472320

[33] Vincent J-L, Zapatero DC (2008) The role of hypotension in the development of acute renal failure. Nephrol Dialysis Transplantation 24:337–338. 10.1093/ndt/gfn679 10.1093/ndt/gfn679 19075191

[34] Zhang JJY, Lee KS, Voisin MR et al (2020) Awake craniotomy for resection of supratentorial glioblastoma: a systematic review and meta-analysis. 10.1093/noajnl/vdaa111. Neurooncol Adv 2:10.1093/noajnl/vdaa111 PMC7542985 33063012

[35] Takami H, Khoshnood N, Bernstein M (2021) Preoperative factors associated with adverse events during awake craniotomy: analysis of 609 consecutive cases. J Neurosurg 134:1631–1639. 10.3171/2020.4.JNS20378 32590355 10.3171/2020.4.JNS20378

[36] Voisin MR, Sasikumar S, Zadeh G (2021) Predictors of survival in elderly patients undergoing surgery for glioblastoma. 10.1093/noajnl/vdab083. Neurooncol Adv 3:10.1093/noajnl/vdab083 PMC8331047 34355171

[37] Mosteiro A, Pedrosa L, Ferrés A et al (2022) The Vascular Microenvironment in Glioblastoma: a Comprehensive Review. Biomedicines 10:1285. 10.3390/biomedicines10061285 35740307 10.3390/biomedicines10061285 PMC9219822

[38] Tatla AS, Justin AW, Watts C, Markaki AE (2021) A vascularized tumoroid model for human glioblastoma angiogenesis. Sci Rep 11:19550. 10.1038/s41598-021-98911-y 34599235 10.1038/s41598-021-98911-y PMC8486855

[39] Maïer B, Fahed R, Khoury N et al (2019) Association of blood pressure during thrombectomy for Acute Ischemic Stroke with Functional Outcome: a systematic review. Stroke 50:2805–2812. 10.1161/STROKEAHA.119.024915 31462188 10.1161/STROKEAHA.119.024915

[40] Custodis F, Schirmer SH, Baumhäkel M et al (2010) Vascular pathophysiology in response to increased heart rate. J Am Coll Cardiol 56:1973–1983. 10.1016/j.jacc.2010.09.014 21126638 10.1016/j.jacc.2010.09.014

